# 国家人体生物监测项目中化学污染物靶向定量分析的实验室质量控制策略

**DOI:** 10.3724/SP.J.1123.2024.11022

**Published:** 2025-06-08

**Authors:** Yanwei YANG, Xu ZHANG, Xiao LIN, Qi SUN, Hui FU, Yifu LU, Tian QIU, Zhuona ZHANG, Linna XIE, Haijing ZHANG, Miao ZHANG, Xiaojian HU, Yingli QU, Feng ZHAO, Yuebin LYU, Ying ZHU, Xiaoming SHI

**Affiliations:** 1.中国疾病预防控制中心环境与健康相关产品安全所，北京 100021; 1. National Institute of Environmental Health，Chinese Center for Disease Control and Prevention，Beijing 100021，China; 2.中国疾病预防控制中心环境与 人群健康重点实验室，北京 100021; 2. China CDC Key Laboratory of Environment and Population Health，Beijing 100021，China; 3.中国疾病预防控制中心，北京 102206; 3. Chinese Center for Disease Control and Prevention，Beijing 102206

**Keywords:** 国家人体生物监测项目, 质量控制, 方法验证, 实验空白, 综述, China National Human Biomonitoring Program （CNHBP）, quality control, method verification, experimental blank, review

## Abstract

国家人体生物监测项目于2016年正式启动，该项目的目标是通过对我国人群进行现场流行病学调查，并监测人体生物样本中的环境污染物，来获取具有代表性的环境污染物在普通人群中的暴露负荷数据，为政府制定环境污染防控政策和采取相应干预措施提供重要的科学依据。为了获取人群生物样本中准确且可比的化学污染物数据，在国家人体生物监测项目的框架下，实验室从分析方法、实验空白到检测过程，对化学污染物的靶向定量分析实施了多维度的质量控制措施。项目的质量控制程序分为两个阶段进行：（1）检测前阶段中生物监测分析方法的验证、空白的筛查与控制；（2）检测阶段中大规模样本分析过程的质量控制。应用于国家人体生物监测项目的分析方法均需经过验证程序，以全面评估方法的性能，验证的重点包括方法检出限和方法定量限、基质效应、稳定性及残留与稀释。所有监测指标均需通过空白筛查程序，以识别、消除或降低实验中的空白干扰，确保每批次的空白测定结果均低于方法检出限。实验室采用内部质量控制与外部质量控制相结合的方式，具体措施主要包括：（1）10类监测指标的方法验证及检测过程均使用包括美国国家标准与技术研究院（NIST）、欧洲标准局（ERM）及中国标准物质中心在内生产的生物基质标准物质，以确保方法的准确性及溯源性；（2）15类监测指标使用商品质控样和内部质控样，以评价测试过程的稳定性；（3）9类共60项监测指标参与德国外部质量评估计划（G-EQUAS），并取得了满意的结果；（4）15类监测指标使用盲样。多维度的质量控制措施为生成高质量的生物监测数据提供了专业支撑。

## Abstract (en)

The China National Human Biomonitoring Program （CNHBP） was launched in 2016. The program aims to obtain representative exposure data of environmental pollutants in the general population by carrying out field epidemiological surveys of Chinese population and monitoring of environmental pollutants in human biological tissues. This work will provide a scientific basis for the government to formulate environmental pollution prevention and control policies. One of the objectives of human biomonitoring is to provide accurate and comparable data of chemical pollutants in human biological samples. Multi-dimensional quality control measures are implemented for the targeted quantitative analysis of chemical pollutants from the analysis method， experimental blank and analysis processes. The quality control procedures are divided into two stages： （1） focusing on the verification of biomonitoring analysis method， blank screening and control in the pre-detection stage； （2） the quality control of the large-scale sample analysis process in the detection stage. Analysis methods used in CNHBP need to be validated to evaluate the performance and practicability， with emphasis on method detection limit （MDL） and method quantification limit （MQL）， matrix effects， stability， and residue and dilution. Blank screening procedures are required for all monitoring indicators to identify， eliminate or reduce blank interference， and the blank value of each batch should be less than the MDL. The laboratory adopts a combination of internal and external quality control measures， the measures mainly include： （1） method validation and detection process of the 10 types of monitoring indicators all used biological matrix reference materials produced by the National Institute of Standards and Technology （NIST）， European Reference Materials （ERM） and China Center for Reference Materials， to ensure the accuracy and traceability of the methods； （2） commercial quality control samples and internal quality control samples were used to evaluate the stability of the testing process for the 15 types of monitoring indicators； （3） a total of 60 monitoring indicators of the nine categories participated in the German external quality assessment scheme for analyses in biological materials （G-EQUAS） and achieved satisfactory results； （4） 15 types of monitoring indicators were tested with blind samples. Overall， multi-dimensional quality control measures provide professional support for generating high-quality biomonitoring data.

化学品已无处不在，据统计，截至2024年，全球登记的化学品数量已超过2.79亿。根据全球化学品展望^［1］^的数据，世界各个区域的化学品生产、使用及贸易量均呈增长趋势。在推动人类社会发展进步的同时，一些有毒有害化学品也给人类和生态环境带来了不可预知的安全、健康和环境风险，特别是那些具有环境持久性、长距离传输性、生物累积性和毒性特征的化学品，受到世界各国的持续关注。识别、控制和防止人群暴露于有毒有害化学品，构成了环境与健康研究工作的基石。人体生物监测（以下简称生物监测）是评估不同暴露来源和途径下人体摄入化学品的综合测量手段，是化学品与人群健康研究的重要方法之一，同时也是国际公认的人群化学品暴露评估的金标准。生物监测数据在确认环境暴露对健康的影响以及验证公共卫生政策方面起到了至关重要的作用。帕尔马环境与健康宣言^［2］^、俄斯特拉瓦夫第六次部长级环境与健康宣言^［3］^和世界卫生组织化学品管理路线图^［4］^等国际协议中均指出，应将促进生物监测应用列为化学品安全领域的优先事项。目前，世界上许多国家和地区均开展了以人群为基础的生物监测研究、调查及实践工作，一些国家亦在国家层面制定并实施了国家人体生物监测计划，如德国环境调查（German Environmental Survey，GerES，1985）^［5］^、美国国家生物监测项目（National Biomonitoring Program，NBP，1999）^［6］^、比利时弗兰斯环境与健康调查（Flemish Environment and Health Survey，FLEHS，2002）^［7］^、加拿大健康测量调查（Canadian Health Measures Survey，CHMS，2006）^［8］^以及欧盟人体生物监测倡议（European Human Biomonitoring Initilative，HBM4EU，2016）^［9］^等。我国的生物监测工作起源于20世纪50年代的职业人群暴露监测。1981年，以北京市区为现场，我国参与了全球环境监测系统的生物监测。尽管多年来一些研究机构和学者开展了环境污染物在普通人群中的暴露水平研究^［10］^，但全国范围内系统化、规范化、持续性的生物监测工作体系尚未建立起来。直至2016年，我国正式启动国家人体生物监测项目，该项目旨在通过现场流行病学调查和人体生物样本中环境污染物的监测，获得具有代表性的环境污染物在普通人群中的暴露负荷数据，从而为政府制定环境污染防控政策和采取相应干预措施提供科学依据。

获取准确的生物监测数据是国家人体生物监测项目的重要目标之一，也是制定公共卫生政策的重要数据基础。然而，环境暴露因素复杂多样，化学污染物种类数量繁杂，生物样本基质特殊且个体差异大，监测指标多属于痕量及超痕量水平，这些因素均对实验分析质量构成了严峻挑战。生物监测涉及多学科、多阶段的协同合作，在整个监测过程中制定并遵循严格的质量控制策略，是获取准确且可比的生物监测数据的前提，这一做法也受到了世界各国生物监测工作的广泛关注。实验分析过程中存在多种影响因素，包括人员、仪器设备、试剂材料、方法、环境及检测过程等，这些因素都可能给实验结果带来不确定性。质量控制是针对上述影响因素所采取的预防和控制措施，从分析角度而言，质量控制可分两个阶段：一是分析方法评价，二是样本检测过程的质量控制与评价。分析方法评价通常通过方法验证来完成，评估的指标包括方法检出限及定量限、准确度、精密度、线性范围、基质效应、空白值、干扰情况以及稳定性等；样本检测过程中的质量控制分为内部质量控制和外部质量控制。内部质量控制由实验室内部组织完成，重点关注校准曲线、空白样品、标准参考物质、质量控制样品及平行样等程序措施的运用与评估，以此来监控检测过程。外部质量控制由实验室外部机构组织完成，一般包括外部质量评价、实验室间比对以及盲样（实验人员未知）测试等，旨在提高检测质量并发现潜在问题。当前，生物监测主要采用色谱-质谱联用技术和光谱-质谱联用技术，其中多数监测指标涉及痕量乃至超痕量分析。方法性能的不稳定及污染问题是导致分析误差的主要因素。因此，提升方法性能并采取有效措施来降低空白污染，是确保实验结果准确性、精密性和可追溯性的关键所在。

截至2024年，国家人体生物监测项目已涵盖15类共233项监测指标。为保证生物样本中化学污染物靶向定量分析的质量，项目从分析方法、实验空白到检测过程，实施了多维度的质量控制措施，旨在最大限度地减少分析误差。项目的质量控制程序分为两个阶段：第一阶段是检测前阶段，包括分析方法验证、空白筛查与控制；第二阶段是检测阶段，包括样本检测过程中所需内部质控样品的制备测试、参与外部质量评估计划和实验室间比对，以及在大规模样本检测过程中进行的内部质控样品和盲样的测定。在项目样本测定过程中，项目组根据分析指标和样品批次大小，将盲样插入到样本分析批次中；实验室内依据分析程序，同步将标准参考物质和质控样品插入到样本分析批次中，与样品一起进行处理和分析，根据样本量的大小鼓励实验室进行双样分析。本文重点结合国家人体生物监测项目，介绍本实验室在质量控制策略方面的应用实践。

## 1 生物监测分析方法的验证

在分析生物样本之前，对定量分析方法进行验证不仅是实验室建立可靠分析系统的基础，也是分析流程中不可或缺的一环，然而这一步骤在实际工作中往往被忽视或简化。我国《化学分析方法验证确认和内部质量控制要求》（GB/T 32465-2015）^［11］^对方法验证确认的内容提出了通用要求。此外，美国^［12］^和欧盟^［13］^分别在2018年和2022年发布了相关指南，均针对生物样本中的分析方法验证及分析方法在生物样本中的应用提出了建议和指导。完整的数据验证对于全面评估分析方法、指导方法应用及确保数据质量具有重要的指导意义。表1列出了国家人体生物监测项目中已实施的监测指标及其分析方法。在应用这些方法之前，本实验室对其适用性、空白值、方法检出限（MDL）与方法定量限（MQL）、线性关系及校准范围、准确度、精密度、稳定性以及基质效应等一系列方法学特性进行了系统验证。基于这些验证结果，我们编制了实验室作业指导书，以规范生物样本的分析工作流程。在生物样本的分析方法验证过程中，相较于其他方法学特性，应特别关注MDL和MQL、基质效应、稳定性及稀释与残留。

**表 1:** 国家人体生物监测项目中已实施的监测指标及其分析方法

Chemicals	Analytes	Analytical methods
Metals and metalloids^［14］^	20 substances in human blood and urine， including arsenic （As）， barium （Ba）， cadmium （Cd）， chromium （Cr）， cobalt （Co）， copper （Cu）， lead （Pb）， mercury （Hg）， manganese （Mn）， molybdenum （Mo）， nickle （Ni）， selenium （Se）， stannum （Sn）， stibium （Sb）， strontium （Sr）， silver （Ag）， thallium （Tl）， titanium （Ti）， vanadium （V）， zinc （Zn）	ICP-MS
Ionic compounds	four substances in human urine， including calcium （Ca）， fluoride （F）， magnesium （Mg）， phosphate （P）	IC
Arsenic species	six substances in human urine， including arsenous acid （As-Ⅲ）， arsenic acid （As-V）， arsenobetaine （AsB）， arsenocholine （AsC）， dimethylarsinic acid （DMA）， monomethylarsonic acid （MMA）	HPLC-ICP-MS
Phthalate （PAE） metabolites^［15］^	15 substances in human urine， including mono-methyl phthalate （MMP）， mono-ethyl phthalate （MEP）， mono-isobutyl phthalate （MiBP）， mono-*n*-butyl phthalate （MnBP）， mono-benzyl phthalate （MBzP）， mono-ethlyhexyl phthalate （MEHP）， mono-cyclohexyl phthalate （MCHP）， mono-ethyl oxohexyl phthalate （MEOHP）， mono-ethyl hydroxyhexyl phthalate （MEHHP）， mono-octyl phthalate （MOP）， mono-isodecyl phthalate （MDP）， mono-isononyl phthalate （MNP）， mono-（2-ethyl-5-carboxypentyl） phthalate （MECPP）， mono-（carboxyoctyl） phthalate （MCOP）， mono-（2-carboxymethylhexyl） phthalate （MCMHP）	UPLC-MS/MS
Bisphenol compounds^［16，17］^	nine substances in human urine， including bisphenol A （BPA）， bisphenol B （BPB）， bisphenol C （BPC）， bisphenol F （BPF）， bisphenol S （BPS）， bisphenol AF （BPAF）， bisphenol AP （BPAP）， tetrachlorobisphenol A （TCBPA）， tetrabromobisphenol A （TBBPA）	UPLC-MS/MS
Polycyclic aromatic hydrocarbon （PAH） metabolites^［18］^	12 substances in human urine， including 1-naphthol （1-NAP）， 2-naphthol （2-NAP）， 2-hydroxyfluorene （2-FLU）， 3-hydroxyfluorene （3-FLU）， 9-hydroxyfluorene （9-FLU）， 1-hydroxyphenanthrene （1-PHE）， 2-hydroxyphenanthrene （2-PHE）， 3-hydroxyphenanthrene （3-PHE）， 4-hydroxyphenanthrene （4-PHE）， 1-hydroxypyrene （1-PYR）， 3-hydroxychrysene （3-CHR）， 6-hydroxychrysene （6-CHR）	UPLC-MS/MS， HRGC-MS
Volatile organic compound metabolites^［19］^	15 substances in human urine， including *N*-acetyl-*S*-（2-hydroxypropyl）-L-cysteine （2HPMA）， 2-methylhippuric acid （2MHA）， *N*-acetyl-*S*-（3-hydroxypropyl）-L-cysteine （3HPMA）， 3-methylhippuric acid+4-methylhippuric acid （3MHA+4MHA）， *N*-acetyl-*S*-（2-carbamoylethyl）-L-cysteine （AAMA）， *N*-acetyl-*S*-（*N*-methylcarbamoyl）-L-cysteine （AMCC）， *N*-acetyl-*S*-（benzyl）-L-cysteine （BMA）， *N*-acetyl-*S*-（2-carboxyethyl）-L-cysteine （CEMA）， *N*-acetyl-*S*-（2-cyanoethyl）-L-cysteine （CYMA）， *N*-acetyl-*S*-（3，4-dihydroxybutyl）-L-cysteine （DHBMA）， *N*-acetyl-*S*-（2-hydroxyethyl）-L-cysteine （HEMA）， mandelic acid （MA）， *trans*， *trans*-muconic acid （MU）， phenylglyoxylic acid （PGA）， *N*-acetyl-*S*-（phenyl）-L-cysteine （S-PMA）	UPLC-MS/MS
Pesticides and their metabolites^［20］^	eight substances in human urine， including para-nitrophenol （PNP）， 3，5，6-tricholor-2-pyridinol （TCPY）， 3-phenoxy benzoic acid （3-PBA）， 4-fluoro-3-phenoxy benzoic acid （4F-3-PBA）， *cis*-dibromovinyl-dimethylcyclopropane carboxylic acid （*cis*-DCCA）， *trans*-dichlorovinyl-dimethylcyclopropane carboxylic acid （*trans*-DCCA）， 2，4-dicholorphenoxyacetic acid （2，4-D）， 2，4，5-tricholorphenoxyacetic acid （2，4，5-T）	UPLC-MS/MS
Antibiotics^［21］^	43 substances in human urine， including azithromycin， clarithromycin， roxithromycin， tilmicosin， erythromycin， chlortetracycline， tetracycline， doxycycline， oxytetracycline， ofloxacin， enrofloxacin， pefloxacin， difloxacin， lomefloxacin， sarafloxacin， besifloxacin， formylciprofloxacin， 8-fluoro gatifloxacin， desmethylofloxacin， sulfamethazine， sulfamethoxazole， sulfadiazine， trimethoprim， sulfameter， sulfaquinoxaline， sulfachloropyridazine， clenbuterol， sulfamonomethoxine， 4-（3′-methylphenyl）amino-3-pyridinesulfonamide， sulfalene， sulfaclozine， ceftiofur， cefaclor， cefotaxime， ampicillin， penicillin V， penicillin G， cefquimide， quinocetone， lincomycin， terbutaline， salbutamol， ractopamine	UPLC-MS/MS
Neonicotinoid insecticide	12 substances in human urine， including deet， acetamiprid-*N*-desmethyl， 3-（diethylcarbamoyl） benzoic acid， *N*-ethyl benzamid-3-carboxylate， *N*，*N*-diethyl-3-（hydroxymethyl） benzamide， imidacloprid， admire， acetamiprid， clothianidin， saichongan， thiacloprid， hydroxyimidacloprid	UPLC-MS/MS
Self-care and consumer product chemicals^［22］^	12 substances in human urine， including methyl paraben （MeP）， ethyl paraben （EtP）， propyl paraben （PrP）， butyl paraben （BuP）， 4-hydroxy benzophenone （4-OHBP）， benzophenone-1 （BP-1）， benzophenone-2 （BP-2）， benzophenone-3 （BP-3）， benzophenone-8 （BP-8）， benzyl paraben （BzP）， triclsan （TCS）， triclocarban （TCC）	UPLC-MS/MS
Per- and polyfluoroalkyl substances^［23］^	18 substances in human serum， including perfluorobutanoic acid （PFBA）， perfluoropentanoic acid （PFPeA）， perfluorohexanoic acid （PFHxA）， prfluoroheptanoic acid （PFHpA）， perfluorooctanoic acid （PFOA）， perfluorononanoic acid （PFNA）， perfluorodecanoic acid （PFDA）， perfluoroundecanoic acid （PFUnDA）， perfluorododecanoate acid （PFDoDA）， perfluorotridecanoate acid （PFTriDA）， perfluorotetradecanoic acid （PFTeDA）， perfluorobutane sulfonate （PFBS）， perfluorohexanesulfonate （PFHxS）， perfluoroheptanesulfonate （PFHpS）， perfluorooctane sulfonate （PFOS）， 4∶2 chlorinated polyfluorinated ether sulfonate （4∶2 Cl-PFESA）， 6∶2 chlorinated polyfluorinated ether sulfonate （6∶2 CI-PFESA）， 8∶2 chlorinated polyfluorinated ether sulfonate （8∶2 Cl-PFESA）	UPLC-MS/MS
Tobacco metabolites^［24］^	two substances in human serum， including cotinine and hydroxycotinine	UPLC-MS/MS
Organochlorines^［25］^	43 substances in human serum， including *α*-hexachlorocyclohexane， *α*-chlordane， aldrin， *β*-hexachlorocyclohexane， *δ*-hexachlorocyclohexane， dieldrin， endosulfan 1， endosulfan 2， endrin， *γ*-hexachlorocyclohexane， *γ*-chlordane， heptachlor， heptachlor epoxide A， heptachlor epoxide B， hexachlorobenzene， isodrin， methoxychlor， mirex， 1，1-dichloro-2，2-bis（2，4′-dichlorophenyl）ethane （*o*，*p*′-DDD）， 1，1-dichlor-2，2-bis（4-chlor-phenyl）ethanep （*p*，*p*′-DDD）， 2-（2-chlorophenyl）-2-（4-chlorophenyl）-1，1-dichloroethylene （*o*，*p*′-DDE）， 1，1-dichloro-2，2-bis（4-chorophenyl）ethylene （*p*，*p*′-DDE）， 1，1，1-trichloro-2-（2-chlorophenyl）-2-（4-chlorophenyl）ethane （*o*，*p*′-DDT）， 1，1-（2，2，2-trichloroethyliden）bis-（4-chlorobenzol）（*p*，*p*′-DDT）， oxychlordane， 2，2′，4，5，5′-pentachlorobiphenyl （PCB101）， 2，3，3′，4，4′-pentachlorobiphenyl （PCB105）， 2，3，4，4′，5-pentachlorobiphenyl （PCB114）， 2，3′，4，4′，5-pentachlorobiphenyl （PCB118）， 2′，3，4，4′，5-pentachlorobiphenyl （PCB123）， 3，3′，4，4′，5-pentachlorobiphenyl （PCB126）， 2，2′，3，4，4′，5′-hexachlorobiphenyl （PCB138）， 2，2′，4，4′，5，5′-hexachlorobiphenyl （PCB153）， 2，3，3′，4，4′，5-hexachlorobiphenyl （PCB156）， 2，3，3′，4，4′，5′-hexachlorobiphenyl （PCB157）， 2，3′，4，4′，5，5′-hexachlorobiphenyl （PCB167）， 3，3′，4，4′，5，5′-hexachlorobiphenyl （PCB169）， 2，2′，3，4，4′，5，5′-heptachlorobiphenyl （PCB180）， 2，3，3′，4，4′，5，5′-heptachlorobiphenyl （PCB189）， 2，4，4′-trichlorobiphenyl （PCB28）， 2，2′，5，5′-tetrachlorobiphenyl （PCB52）， 3，3′，4，4′-tetrachlorobiphenyl （PCB77）， 3，4，4′，5-tetrachlorobiphenyl （PCB81）	GC-MS/MS
Brominated flame retardants^［26］^	14 substances in human serum， including 2，2′，4′-tribromodiphenyl ether （BDE-17）， 2，4，4′-tribromodiphenyl ether （BDE-28）， 2，2′，4，4′-tetrabromodiphenyl ether （BDE-47）， 2，3′，4，4′-tetrabromodiphenyl ether （BDE-66）， 2，3′，4′，6-tetrabromodiphenyl ether （BDE-71）， 2，2′，3，4，4′-pentabromodiphenyl ether （BDE-85）， 2，2′，4，4′，5-pentabromodiphenyl ether （BDE-99）， 2，2′，4，4′，6-pentabromodiphenyl ether （BDE-100）， 2，2′，3，4，4′，5′-hexabromodiphenyl ether （BDE-138）， 2，2′，4，4′，5，5′-hexabromodiphenyl ether （BDE-153）， 2，2′，4，4′，5，6′-hexabromodiphenyl ether （BDE-154）， 2，2′，3，4，4′，5′，6-heptabromodiphenyl ether （BDE-183）， 2，3，3′，4，4′，5，6-heptabromodiphenyl ether （BDE-190）， 2，2′，3，3′，4，4′，5，5′，6，6′-decabromodiphenyl ether （BDE-209）	HRGC-MS

### 1.1 MDL和MQL

生物样本中的目标分析物通常以痕量或超痕量水平存在。MDL和MQL是衡量方法灵敏度的关键指标。MDL是指样品中能够被定性检测出来而无需准确定量的最低含量，其评估方法较多，如空白标准偏差法、信噪比法、逐步稀释法以及仪器灵敏度法等。美国于2016年发布MDL的定义及确定程序（Definition and Procedure for the Determination of the Method Detection Limit）^［27］^中详细阐述了MDL的定义及其确定流程。该流程要求在不同时间点分批次处理至少7个加标样品和7个空白样品。对于加标样品，通过测定结果的3倍标准偏差来计算MDL（表示为MDL_s_）；对于均有检出的空白样品，则采用空白结果的3倍标准偏差加上空白均值的方法来计算MDL（表示为MDL_b_）。将MDL_s_与MDL_b_进行比较，以数值较大者作为最终的MDL。该文件特别强调，MDL的评估与确定过程必须涵盖样品预处理的所有步骤，并考虑实验空白所产生的影响。部分方法过于追求“检测极限”，却忽视了预处理步骤及空白对MDL评估的重要性，这往往会导致MDL被低估。例如，在检测邻苯二甲酸单异丁酯（MiBP）、邻苯二甲酸单正丁酯（MnBP）和邻苯二甲酸单乙基己基酯（MEHP）这3种邻苯二甲酸酯代谢物的过程中，常受到难以消除的空白干扰。比较两种方法确定的MDL，依据美国发布的MDL定义及确定程序^［27］^计算的MDL要高于通过信噪比法计算的MDL。具体而言，这3种邻苯二甲酸酯代谢物依据上述两种方法确定的MDL分别为0.85 µg/L和0.05 µg/L（MiBP）、0.80 µg/L和0.04 µg/L（MnBP）、0.50 µg/L和0.05 µg/L（MEHP）^［15］^。由此可见，采用不同方法评估的MDL之间存在一个数量级的差异，因此选择合理的MDL评估方法对于确保检测方法的准确性至关重要。

MQL是指样品中能够被定量分析的最低含量，它应当具有可接受的准确度和精密度。例如，美国发布的工业生物分析方法验证指南（Bioanalytical Method Validation Guidance for Industry）^［12］^中，建议MQL的准确度为80%~120%，精密度不超过20%。一般而言，MQL应为校准曲线的最低浓度点。

### 1.2 基质效应

生物样本基质复杂，在应用质谱技术，尤其是液相色谱-质谱联用技术建立分析方法时，基质效应是一个普遍存在的问题。这种基质效应可能对方法学特性和分析结果产生整体或局部的影响。例如，尿液对多个苯系物代谢物表现出明显的基质抑制作用，过强的抑制作用会降低分析信号，进而影响方法的灵敏度。美国疾病预防控制中心实验室的数据显示，约有5%的样品因基质效应而严重干扰了定量分析的结果。因此，美国食品和药品监督管理局在工业生物分析方法验证指南^［12］^中，明确规定了在使用液相色谱-质谱联用技术建立生物样本分析方法时，需考察基质效应对目标分析物测定结果的影响。欧盟同样也在生物分析方法验证和研究样品分析指南（Guideline M10 on Bioanalytical Method Validation and Study Sample Analysis）^［13］^中细化了基质效应的评判标准。我国的《生物样品定量分析方法验证指导原则》^［28］^也明确指出，在采用质谱方法进行生物样品分析时，需要对基质效应进行考察。目前，基质效应的评价通常采用柱后灌注法和提取后加入法^［29］^。在国家人体生物监测项目中，分析方法多采取向样品提取液中加入标准品的方式，并比较不同基质中标准曲线的斜率来评估基质效应；同时，采用与目标分析物一一对应的同位素内标来进行补偿或校正，以消除基质效应的影响。例如，在采用固相萃取-超高效液相色谱-串联质谱（SPE-UPLC-MS/MS）法测定尿液中多种农药代谢物的过程中，选取6个不同来源的尿液样本，按照既定的样本前处理方法进行处理。随后，以6个提取尿液为基质，分别配制不同浓度的基质匹配混合标准溶液；同时以甲醇为溶剂，配制不同浓度的溶剂混合标准溶液，最后通过计算提取尿液基质标准曲线的斜率与甲醇溶剂标准曲线的斜率的比值来评估方法的基质效应。在生物样本分析中，向样品中加入与目标分析物一一对应的内标是抵消基质效应最常用的方法。由于内标与目标分析物在处理和分析过程中具有相同的损失率、离子增强和抑制程度，因此它们的比值保持不变。该方法可以提高定量分析的准确度、精密度以及方法的稳健性。同位素内标被视为最为理想的内标选择，其中由于价格相对较低，氘代内标相较于^13^C和^15^N内标更为常用。然而，氘代内标分子结构中的氘原子容易与氢原子发生交换反应，这可能会导致分析结果出现偏差，甚至可能引发空白污染。因此，在使用氘代内标之前，应进行充分的评估以确保其适用性。例如，在采用液液萃取-气相色谱-高分辨双聚焦磁质谱（LLE-GC-HRMS）法测定尿液中的多环芳烃羟基代谢物的研究中^［18］^，配制1-羟基萘（1-NAP）和2-羟基萘（2-NAP）的氘代内标溶液，并将其冷藏保存半年。然而，由于氘原子与氢原子之间发生了交换反应，导致在实验空白中检测到了明显的1-NAP和2-NAP信号。

### 1.3 稳定性

生物样本通常通过冷藏或冷冻的方式进行保存。样本的制备及分析过程会伴随温度、基质、时间、存储条件的变化，但这些变化不应造成目标化合物含量的改变，或目标化合物含量偏离情况应在可接受范围内。稳定性评价通常涵盖以下几个方面：目标化合物在储备液和工作液中的稳定性、从冷冻状态变至样品制备温度时的冻融稳定性、样品制备期间的稳定性以及待测样品溶液在进样期间的稳定性等。试验条件应尽量与样品保存、预处理及实际检测条件保持一致。不同化合物在不同条件下的稳定性各异。例如，对尿液中邻苯二甲酸酯代谢物的稳定性进行研究时发现，在‒20 ℃和‒70 ℃条件下，尿液样本中的邻苯二甲酸酯代谢物可以稳定保存60 d。然而，尿液样本经过处理后，在4 ℃和‒20 ℃条件下保存7 d，12种邻苯二甲酸酯代谢物均出现不同程度的降解，其中邻苯二甲酸单-（2-乙基-5氧己基）酯（MEOHP）的降解最为明显，保存仅1 d后其峰面积就下降了25.7%^［15］^。这一实验结果提示，样品处理完成后应尽快进行分析。

### 1.4 残留与稀释

不同生物样本的暴露水平存在较大差异。例如，苯、甲苯、二甲苯等苯系物常出现在烟草烟雾中，导致吸烟人群的暴露水平明显高于非吸烟人群^［19］^。此外，两种苯代谢物（反，反-黏糠酸和苯巯基尿酸）以及3种二甲苯代谢物在吸烟者与非吸烟者尿液中的含量差异具有统计学意义。当样品中目标分析物的含量较高时，这些物质在检测仪器中的残留可能会对后续样品的测定结果产生干扰。为了验证方法的有效性，实验人员在分析高浓度样品或标准溶液后，应注射空白样品以评估残留影响。

美国在工业生物分析方法验证指南^［12］^中建议，目标分析物的残留量不应超过MQL的20%。若残留无法避免，则应考虑采取特殊措施，例如在对高浓度样品进行分析后应插入空白样品，以消除残留带来的影响。当样品中目标分析物的浓度超出校准曲线范围时，应对样品进行稀释或降低取样量后重新制备，且稀释过程不应影响方法的准确度和精密度。在方法验证过程中，可以向基质中加入一定量的目标分析物来制备高浓度样品，并通过计算稀释后样品的准确度和精密度来对其进行评价。

## 2 生物监测中空白的挑战与控制

实验空白构成了生物监测中痕量或超痕量分析的一大难点与挑战。许多化学物质在环境中普遍存在，即便采用最先进的实验室设施与分析方法，空白污染问题也难以完全避免。一般而言，生物样本分析测定的全过程均可能引入污染，污染来源常概括为以下几个方面：（1）实验环境污染和实验残留；（2）实验过程用水、酶及各种纯度不足的试剂；（3）实验耗材中的杂质；（4）实验器皿及仪器设备残留；（5）实验人员自身带入或因操作水平经验不足而导致的潜在污染。

### 2.1 空白污染的来源

在国家人体生物监测项目中，多种监测指标均存在实验空白污染问题，但不同监测指标的实验空白污染来源有所差异。例如，双酚类物质广泛应用于环氧树脂及聚碳酸酯塑料工业中，因其具有内分泌干扰效应而受到了广泛关注，同时双酚类物质也是许多生物监测项目关注的指标。本实验室采用SPE-UPLC-MS/MS技术，对尿液中的双酚类物质进行了测定。在此过程中发现，实验空白污染主要源自实验耗材（如注射器和管尖等）以及局部环境的残留^［30］^。选用不同规格（200 µL、1 mL和5 mL）的管尖和不同品牌的注射器，反复吸取甲醇至少10次来模拟实验过程，并对吸取液进行了测定。结果显示，5 mL管尖中可检出双酚A（BPA）和双酚F（BPF），二者的检出水平分别为0.11~0.36 µg/L和0.11~0.14 µg/L；注射器中可检出BPA，检出水平为0.10~0.34 µg/L；在长时间使用且未及时清洁的局部环境（如通风橱）中开展尿样双酚类物质检测时，空白实验可检出约1 µg/L的BPA。多环芳烃因其致癌性，成为多个生物监测项目重点关注的指标。本实验室采用LLE-GC-HRMS技术，测定了尿液中的多环芳烃羟基代谢物。实验发现，该类指标的实验空白污染主要源于氘代内标的氘-氢交换反应、实验人员的吸烟行为以及实验局部环境的残留^［31］^。实验结果表明，保存了半年时间的1-NAP-d_7_和2-NAP-d_7_氘代内标溶液中出现了明显的1-NAP和2-NAP信号；此外，如果实验人员有吸烟习惯，或者实验室周围环境一定距离内有其他人吸烟，也会导致实验空白中的2-NAP信号显著升高。在使用SPE-UPLC-MS/MS法测定人尿中的全氟及多氟烷基化合物时，甲酸、氨水及人工尿液的使用可能会引起4种全氟羧酸和全氟辛烷磺酸的实验空白污染问题^［32］^。同样地，当采用SPE-UPLC-MS/MS法测定人尿中的个人护理品功效成分时，实验人员使用个人护理用品的行为也可能导致二苯酮-3、三氯生等指标出现实验空白污染现象^［22］^。

### 2.2 空白污染的评估与控制

在实验分析过程中，各种潜在的污染及残留可能会导致不同程度的空白污染问题。生物样本中监测指标的含量通常较低，实验空白的大小及其离散程度对方法学特性和生物样本的测定结果具有较大影响。具体而言，实验空白值越大，评估确定的MDL就会越高，方法的灵敏度也就越低，从而无法满足痕量及超痕量分析的需求。对于含量本就较低的指标而言，这种情况甚至可能导致测定结果失效。识别、消除或有效降低实验空白污染对于准确测定生物样本中的各类监测指标具有重要意义，这也是预分析阶段的重要工作和程序。在国家人体生物监测项目的执行过程中，本实验室在每类监测指标的方法建立阶段便对实验过程中的各种可能因素进行全面筛查，以识别潜在的污染或残留问题。通过选择优化试剂耗材、增加实验环境和仪器设备的清洗维护频次、避免个人护理用品的使用以及加强实验人员的培训与实践等措施来消除或降低空白污染至可接受水平。

随着实验环境、仪器设备、材料及人员等因素的变化，空白污染问题会呈现不确定性。因此，本实验室在进行生物样本分析时，确保每一分析批次均至少包含一个实验空白，用以监控测定过程中可能存在的污染。原则上，实验空白中目标分析物的测定结果应低于MQL；若超出MQL，则样品测定结果将被视为可疑。当样品分析过程中出现空白污染时，应分析污染来源并消除。若无法消除空白污染，则需评估其对样品测定结果的影响，并考虑扣除空白的可能性。在此过程中，可参照《职业人群生物监测方法总则》（GBZ/T 295-2017）^［33］^中规定的空白结果处理原则。若空白对样品测定结果的相对影响率低于最大允许标准，则可从样品测定结果中扣除样品空白；反之，若相对影响率超出该标准，则样品测定结果视为无效，需重新制备样品并进行分析，或者重新评估并确定MDL和MQL。

## 3 大规模生物样本检测过程的质量控制

大规模生物样本检测过程的质量控制多采取内部质量控制与外部质量控制相结合的方式进行。例如，通过参与外部质量评估计划来验证实验室的检测能力，利用标准参考物质来评估测定结果的准确性和溯源性，开展实验室间比对以确保数据可比性，以及使用质控样品和盲样测试来评价检测过程的稳定性。在国家人体生物监测项目的实施过程中，实验室对15类监测指标实施了多项质量控制程序与措施，具体见表2。

**表 2:** 样本检测过程中的质量控制措施

Chemicals	SRM	Quality control sample	G-EQUAS	Interlaboratory comparison	Blind sample
Metals and metalloids	/	√	√	√	√
Ionic compounds	/	√	√	√	√
Arsenic species	√	√	/	√	√
PAE metabolites	√	√	√	√	√
Bisphenol compounds	√	√	√	√	√
PAH metabolites	√	√	√	√	√
Volatile organic compound metabolites	√	√	√	√	√
Pesticides and their metabolites	/	√	√	√	√
Antibiotics	√	√	/	/	√
Neonicotinoid insecticide	/	√	/	/	√
Self-care and consumer product chemicals	√	√	/	√	√
Per- and polyfluoroalkyl substances	√	√	√	√	√
Tobacco metabolites	/	√	/	√	√
Organochlorines	√	√	√	√	√
Brominated flame retardants	√	√	/	√	√

SRM： standard reference material； G-EQUAS： the German external quality assessment scheme for analyses in biological materials； /： no； √： yes.

### 3.1 标准参考物质

标准参考物质是分析测量的溯源基础，在仪器设备校准、分析方法评估、人员考核等多个环节中发挥着不可或缺的作用，同时也是检测过程中质量控制和结果评判的优先选择。目前适用于生物监测的标准参考物质数量有限且昂贵。美国国家标准与技术研究院（National Institute of Standards and Technology，NIST）、欧洲标准局（ERM）和中国国家标准物质中心可提供涵盖部分监测指标的基质标准参考物质，以支持生物样本中特定化学污染物的测量需求。标准物质的特性值一般分为认证值和非认证值。认证值是由权威机构认定的，具有不确定度和溯源性，其在准确度方面具有更高的可信度；非认证值是对真实值的估算，无法完全表征不确定度的来源。NIST标准物质不仅提供认证值，还针对部分指标提供非认证值。我国标准物质均提供认证值。例如，NIST研制的非吸烟人群尿中有机污染物标准物质（SRM 3673 Organic contaminants in non-smokers′urine）提供了10个多环芳烃代谢物指标的认证值，同时还提供了45个其他指标的非认证值，这些指标包括邻苯二甲酸酯类代谢物、环境酚类、苯甲酸酯类、挥发性有机物代谢物、布洛芬及咖啡因等^［34］^。

在国家人体生物监测项目中，有10类指标的方法验证与检测流程采用了NIST、ERM以及中国国家标准物质中心提供的生物基质标准物质，具体见表3。对于具有认证值的标准物质，其测量结果需落在给定的参考范围内；而对于提供了非认证值的标准物质，检测过程中常通过计算标准物质指标的平均回收率来评估检测流程的质量。以尿液中的邻苯二甲酸酯代谢物为例，在检测过程中使用NIST SRM 3672来评价样本检测质量。在为期一年的监测期间，7种监测指标的平均回收率为87.7%~108.3%，相对标准偏差为4.9%~10.9%，具有可接受的准确度和精密度范围。

**表 3:** 人体生物监测项目中标准参考物质的应用情况

Chemicals	Matrix	SRMs
Arsenic species	urine	NIST SRM 2669： arsenic species in frozen human urine GBW 09115： arsenic species in freeze-dry human urine
PAE metabolites	urine	NIST SRM 3672： organic contaminants in non-smokers' urine
Bisphenol compounds	urine	NIST SRM 3673： organic contaminants in non-smokers' urine
PAH metabolites	urine	NIST SRM 3673： organic contaminants in non-smokers' urine
Volatile organic compound metabolites	urine	NIST SRM 3673： organic contaminants in non-smokers' urine
Antibiotics	urine	ERM BCR 503： bovine urine （clenbuterol and salbutamol）
Self-care and consumer product chemicals	urine	NIST SRM 3673： organic contaminants in non-smokers' urine
Per- and polyfluoroalkyl substances	serum	NIST SRM 1957： organic contaminants in non-fortified human serum
Organochlorines	serum	NIST SRM 1957： organic contaminants in non-fortified human serum
Brominated flame retardants	serum	NIST SRM 1957： organic contaminants in non-fortified human serum

GBW： national standard substance； NIST： National Institute of Standards and Technology； ERM： European reference materials.

### 3.2 内部质控样品

在生物样本检测过程中，当标准参考物质无法覆盖所有监测指标时，内部质控样品也可作为一种常用的质量控制手段，用以监控分析系统的稳定性。实验室既可以选用商品化的质控样品，也可以自行制备内部质控样品。以农药及其代谢物指标为例，内部质控样品的制备过程如下：取若干名普通人群志愿者的尿液样本（约1.5 L）于玻璃瓶中混匀，用滤纸过滤，以去除颗粒物；根据预期浓度向尿液样本中添加农药及其代谢物的混合标准溶液，混匀后将样本分装至2.5 mL冻存管中，并置于冰箱中冷冻保存。内部质控样品均为一次性使用，以避免因多次冻融循环而导致的不稳定性问题。在使用前，还需对内部质控样品进行均匀性评估和含量测定，并绘制质控图。在分析批次中插入内部质控样品，并与之同步处理和分析。若每批次的测定结果落在质控图的警告限（±2倍标准偏差）范围内，则分析结果被视为准确可靠；若测定结果位于警告限与控制限（±3倍标准偏差）之间，则表明分析结果的可靠性有所降低，需关注并校正分析系统；一旦测定结果超出控制限，则分析结果不符合要求，应停止测定并查找原因。本实验室在测定尿液中8种农药及其代谢物的过程中，得到的内部质控样品质控图如附图1（www.chrom-China.com）所示。在一年内，实验室共分析了1 900余份尿液样本，每批次分析样本均采用高、低两个加标水平的内部质控样品进行监测。结果显示，在一年间测定82次，8种指标的总体变异系数均低于11.8%，表明分析系统具备可靠的稳定性。

### 3.3 参与外部质量评估计划

外部质量评估计划也称能力测试，通常由认证机构负责组织实施。多个国家和机构已开展了针对生物样本分析的评估计划。例如，德国外部质量评估计划（G-EQUAS）^［35］^、荷兰和瑞典组织的持久性有机污染物全球实验室间评估^［36］^、美国铅和多元素能力验证（LAMP）^［37］^以及欧盟人体生物监测倡议实验室间比较调查和外部质量评估计划（ICI/EQUAS）^［38］^等。这些评估计划可采用多种方式，如采用Z评分模型、指定值、基于参考实验室确定的公议值以及基于参与者确定的公议值等方式。外部质量评估计划的组织者对参与实验室提交的结果进行统计分析后，向各实验室提供性能评估报告。

自2018年起，本实验室定期参与G-EQUAS，参与的指标数量在2018年至2023年期间从9个增长至49个，目前共涵盖了9类共60项指标（详见表4），并取得了令人满意的结果。参与外部质量评估计划可提高实验室对特定指标的检测能力以及提高检测结果的准确性和可比性。然而，单一评估计划的指标覆盖范围有限。例如，采用气相色谱-串联质谱（GC-MS/MS）法可测定血清中的43种有机氯化合物，但G-EQUAS中仅包含其中的11种指标。因此，为了有效提高指标覆盖范围及检测能力，可选择参与多个外部质量评估计划或组织实验室间比对。

**表 4:** 参与G-EQUAS的监测指标

Chemicals	Matrixes	Analytes
Metals and metalloids	blood/urine	As， Cd， Cr， Co， Hg， Mn， Ni， Pb， Zn
Ionic compounds	urine	F， Ca
Phthalate metabolites	urine	MEP， MnBP， MiBP， MBzP， MEHP， 5-OH-MEHP， 5-OXO-MEHP， 5-carboxy-MEPP
Bisphenol compounds	urine	BPA， BPS， BPF
Polycyclic aromatic hydrocarbon metabolites	urine	1-NAP， 2-NAP， 1-PYR
Volatile organic compound metabolites	urine	HA， MHA， MA， S-PMA， 2-HPMA， 3-HPMA， AAMA， AMCC， DHBMA， CEMA， HEMA
Per- and polyfluoroalkyl substances	serum	PFOA， PFOS， PFNA， PFDA， PFHxS， PFHpS， PFBS， FPBA
Pesticides and their metabolites	urine	*cis*-DCCA， *trans*-DCCA， 3-PBA， PNP， TCPY
Organochlorines	serum	*p*，*p′*-DDE， DDT， *α*-HCH， *β*-HCH， *γ*-HCH， PCB-28， PCB-52， PCB-101， PCB-138， PCB-153， PCB-180

### 3.4 盲样

盲样也是外部质量控制的一种常见手段，通常由项目组在样本发放过程中，根据分析指标和样品批次大小，按比例将盲样插入实际样品中，以供实验室同步处理和分析。项目组会对生成的数据进行统计，以提高分析质量并发现潜在问题。以金属及类金属的测定为例，实验室在一年内分析了4 000余份尿液样本，并在测定过程中插入了5个不同水平的盲样。结果显示，17项测定指标的总体变异系数均低于21.0%（图1）。

**图1:**
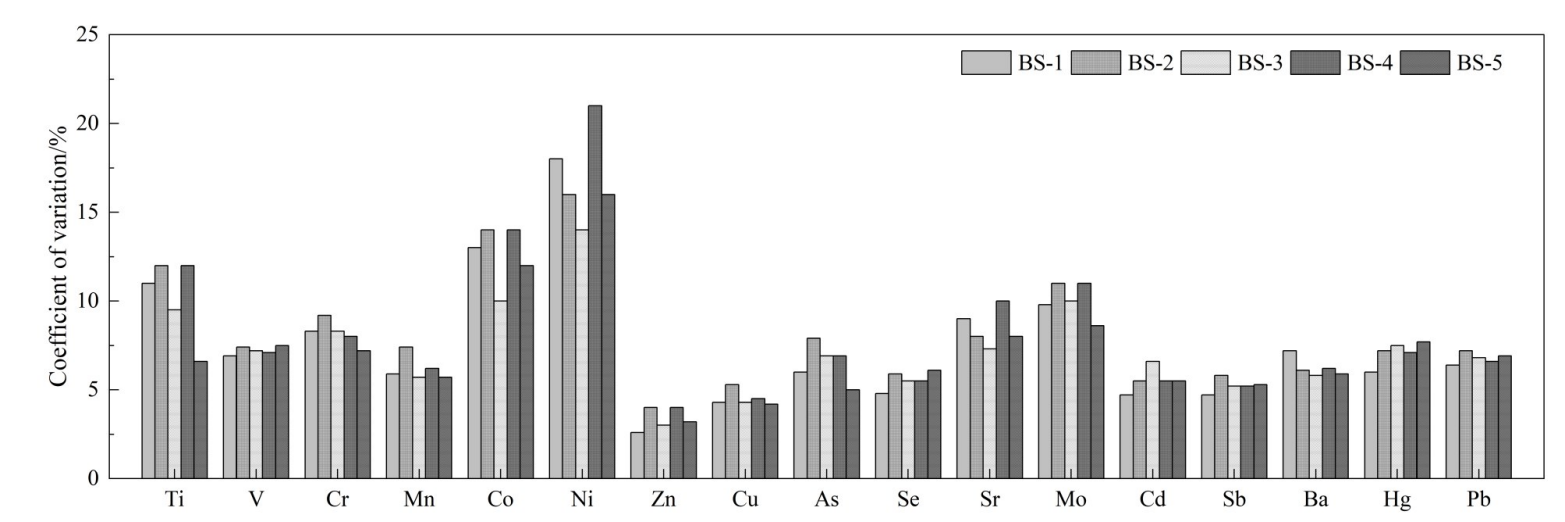
17种金属及类金属测定过程中5个盲样的变异系数

## 4 总结

生物监测作为评估化学品暴露的有效手段，已广泛获得政府部门、科研人员及公众的认可。质控策略与方案作为生物监测的关键组成部分，对于生成高质量数据起到了至关重要的作用。然而，随着生物样本中生物标志物种类的迅速增多以及人体生物样本分析技术的快速发展，越来越多的分析实验室进入生物监测领域。相比之下，生物样本的化学分析面临着诸多挑战。例如，目前尚未构建完善的生物监测标准方法体系，实验空白问题普遍存在，基于人体生物样本的外部质量评估计划和标准参考物质未得到广泛研发，且基质类型及指标覆盖范围均有限。这些因素限制了生物监测数据的质量评估以及不同研究数据之间的可比性。本实验室依托国家人体生物监测项目，在生物样本的靶向定量分析方面实施了广泛的程序措施。针对生物监测的方法、实验空白及检测过程的不同特点，采取相应的程序措施，以协同减少或消除误差。这些程序措施的属性不同，相互补充，为新方法的开发和生成准确、稳定、可比的生物监测数据提供了专业的支持。此外，鉴于监测指标繁多，由此累积的质控数据将为探索分析批间、实验室间以及不同研究间数据的可比性奠定数据基础。
